# Site‐Specific Associations Between CBCT‐Derived Gray Value and Fractal Dimension and DEXA T‐Scores in Postmenopausal Women: A Cross‐Sectional Analytical Study

**DOI:** 10.1002/cre2.70474

**Published:** 2026-10-04

**Authors:** Golnoush Saeidi, Mohammad Mahdi Maleki, Abbas Shokri, Leili Tapak

**Affiliations:** ^1^ Department of Oral and Maxillofacial Radiology, Dental School Hamadan University of Medical Sciences Hamadan Hamadan Iran; ^2^ Student Research Committee, Hamadan University of Medical Sciences Hamadan Iran; ^3^ Dental Implants Research Centre, Dental School, Hamadan University of Medical Sciences Hamadan Hamadan Iran; ^4^ Professor, Dental Implants Research Center, Department of Oral and Maxillofacial Radiology, Dental School Hamadan University of Medical Sciences Hamadan Hamadan Iran; ^5^ Department of Biostatistics, School of Public Health—Modeling of Noncommunicable Diseases Research Center, Institute of Health Sciences and Technologies Hamadan University of Medical Sciences Hamadan Hamadan Iran

## Abstract

**Objective:**

Osteoporosis is a silent systemic skeletal disease characterized by microarchitectural deterioration and increased fracture risk; thus, early case‐finding is essential. Although dual‐energy X‐ray absorptiometry (DEXA) is the reference standard for bone mineral density (BMD) assessment, limited accessibility and cost restrict its use for large‐scale screening. Given that cone‐beam computed tomography (CBCT) is routinely obtained in dental practice, image‐derived parameters—gray value (GV) and fractal dimension (FD)—may provide insight into their association with systemic bone mineral density measured by DEXA in postmenopausal women, without additional radiation exposure.

**Materials and Methods:**

This cross‐sectional study included 63 postmenopausal women aged 50–70 years undergoing dental CBCT (NewTom 3G; standardized acquisition). Six trabecular regions of interest (ROIs) were evaluated: mandibular retromolar, mandibular premolar, mandibular anterior (central), maxillary premolar, maxillary anterior (central), and maxillary tuberosity. GV values were extracted using OnDemand3D‐Dental from standardized 25 × 25‐pixel ROIs positioned to exclude cortical bone and dental structures. FD was calculated in ImageJ using a box‐counting method following Gaussian blur subtraction, binarization, and skeletonization. Lumbar spine and hip BMD were measured by DEXA and categorized according to WHO criteria. Statistical analyses included *t*‐tests/ANOVA, Pearson correlation (*α* = 0.05). Intra‐observer reliability was assessed with intraclass correlation coefficients (ICC).

**Results:**

DEXA classified 49.2% as normal, 34.9% as osteopenic, and 15.9% as osteoporotic. Lumbar assessment identified more non‐normal cases than the hip evaluation. Reliability was good (ICC > 0.70). Although mean GV and FD did not differ significantly between groups, site‐specific GV–FD correlations were observed. Notably, FD at the maxillary premolar correlated with lumbar T‐scores in the non‐normal group (*p* = 0.030).

**Conclusion:**

While GV and FD did not discriminate BMD categories overall, region‐dependent associations between CBCT‐derived parameters and systemic BMD were observed, suggesting potential clinical relevance in dental imaging. These exploratory findings should not be interpreted as diagnostic or screening indicators and require confirmation in larger studies.

## 1 Introduction

Osteoporosis is a systemic skeletal disorder characterized by reduced bone mass and microarchitectural deterioration, leading to increased bone fragility and susceptibility to fracture. Often referred to as a “silent disease,” osteoporosis typically remains asymptomatic until a fracture occurs, which underscores the importance of early detection in asymptomatic individuals during the initial stages of bone loss. Dual‐energy X‐ray absorptiometry (DEXA) is the current gold standard for assessing bone mineral density (BMD); however, it imposes substantial costs and requires specialized equipment, which may limit its accessibility in certain populations (Walker et al. 2020; Winzenberg and Jones 2011).

Several radiographic approaches have been proposed to evaluate bone quality, among which fractal dimension (FD) analysis and gray value (GV) measurement have gained attention. FD analysis quantifies the complexity of trabecular bone microarchitecture using mathematical algorithms, providing an indirect yet highly informative assessment of bone quality (Lucas et al. 2017; Lopes and Betrouni 2009; Tafraouti et al. 2014). On the other hand, GV—representing the mean pixel intensity in a grayscale image—offers a simple, reproducible surrogate for radiodensity in medical images (Lucas et al. 2017; Barngkgei et al. 2014).

The prevalence of osteoporosis increases with age, particularly among postmenopausal women, in whom estrogen deficiency accelerates bone resorption and decreases calcium absorption, significantly elevating the risk of fractures (Marcus and Majumder 1996; Cummings et al. 1985; Lindsay et al. 2001; Paschalis et al. 2016; Gambacciani 2020). Epidemiological studies indicate that nearly 50% of postmenopausal women and 20% of men over 50 years old experience osteoporosis‐related fractures during their lifetime (Adler 2020; Sridevi and Ragi 2016; Pickhardt et al. 2011). Hip and vertebral fractures, in particular, impose substantial socioeconomic and health burdens, and can even be life‐threatening in elderly populations (Black and Rosen 2016; Rachner et al. 2011).

While DEXA remains the reference method for osteoporosis diagnosis, alternative imaging techniques such as cone‐beam computed tomography (CBCT) have emerged in dental and maxillofacial practice. CBCT offers high spatial resolution, lower radiation dose compared to conventional CT, and relatively lower cost, making it a widely used modality in implant planning and other dental applications (De Vos et al. 2009; Bushong 2013; Tsiklakis et al. 2004). Since jaw bones share physiological and structural characteristics with other skeletal sites, it has been hypothesized that CBCT‐derived bone quality indices—such as FD and GV—may serve as surrogates for systemic bone status, and could potentially be explored for their association with systemic bone status during routine dental imaging (Horner et al. 2007; Nackaerts et al. 2008; Ruttimann et al. 1992; Cassetta et al. 2014).

Previous studies have demonstrated significant correlations between FD values from jaw trabecular bone and systemic BMD measured at sites such as the lumbar spine and femoral neck (Tafraouti et al. 2014; Guerra et al. 2017; de Castro et al. 2020). Similarly, GV measurements in CBCT images have been associated with variations in bone density, although results are sometimes affected by imaging parameters, region of interest (ROI) selection, and image processing methods. Notably, both FD and GV analyses can be performed retrospectively on existing CBCT datasets without additional radiation exposure, which is a substantial advantage over other modalities (Cassetta et al. 2014; Shrout et al. 1997). More recent CBCT investigations have continued to examine these relationships: mandibular fractal dimension has shown differences between postmenopausal women with normal and low bone mineral density (Carvalho et al. 2022), cervical‐vertebra gray values have been associated with osteoporosis and osteopenia in postmenopausal women (Slaidina et al. 2022), and a systematic review concluded that CBCT shows reasonable accuracy in predicting low bone mass (Isayev et al. 2023).

The novelty of this study lies in its site‐specific evaluation of the association between CBCT‐derived gray values and fractal dimensions with DEXA T‐scores in postmenopausal women, providing localized insight into associations that complement the generalized bone density assessments performed in previous research. Given the increasing number of edentulous elderly patients requiring dental implants and the critical role of adequate bone quality in implant success, there is a growing need for accessible, non‐invasive, and cost‐effective methods to assess bone density. This study aimed to investigate the relationship between GV and FD measurements obtained from CBCT images and systemic BMD assessed via DEXA in postmenopausal women. Establishing significant associations between these indices may provide preliminary evidence supporting further investigation of CBCT‐derived metrics in relation to systemic bone mineral density. Importantly, this study was designed as a cross‐sectional analytical investigation of statistical associations and was not intended to evaluate diagnostic performance or screening accuracy.

## 2 Materials and Methods

### 2.1 Study Population

This cross‐sectional study included 63 postmenopausal women, aged 50–70 years, who presented to the Oral and Maxillofacial Radiology Department at Hamadan University of Medical Sciences between 2023 and 2025 for CBCT imaging. The study population consisted of postmenopausal women aged between 50 and 70 years who were referred to the radiology department for CBCT imaging for dental purposes unrelated to this research. Menopause was defined as the absence of menstruation for at least 12 consecutive months.

To be eligible, participants were required to be postmenopausal, within the specified age range, and free from systemic conditions affecting bone metabolism. Women were excluded if they had any systemic disease known to influence bone turnover (e.g., cancer, osteomalacia, endocrine disorders such as hyperthyroidism or hyperparathyroidism, severe renal or hepatic failure, or rheumatoid arthritis), a history of non‐traumatic fracture or oophorectomy, or current or previous use of medications known to interfere with bone metabolism (e.g., corticosteroids, hormone replacement therapy, antiresorptive or anabolic agents). Individuals with a history of tobacco use or alcohol consumption were also excluded from the study.

The sample size was calculated using Hulley's formula, assuming an expected correlation coefficient of 0.33, *α* = 0.05, and 90% power, with an additional 10% to account for potential attrition. This yielded a required sample size of 63 participants. Convenience sampling was employed to recruit eligible participants (Figure 1).

**Figure 1:**
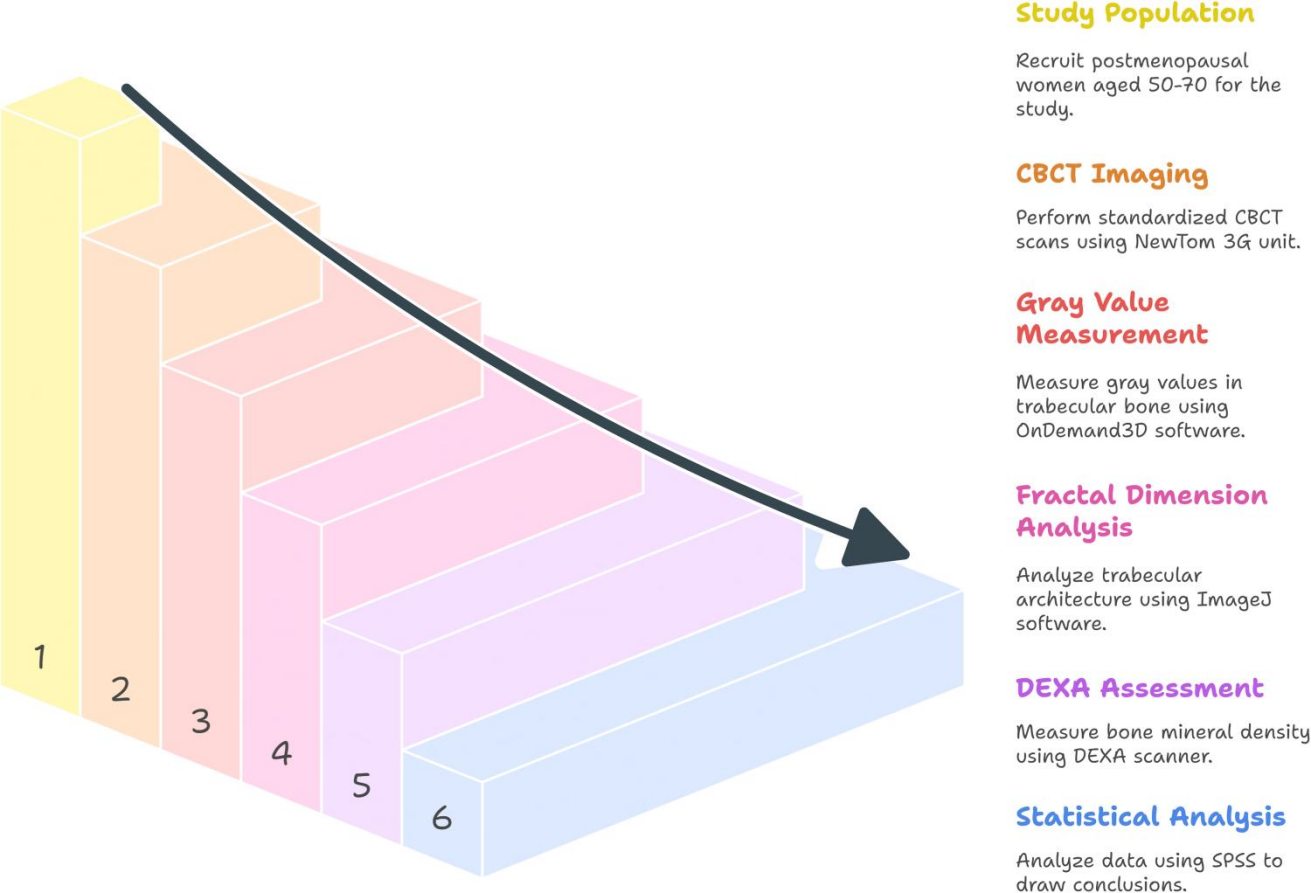
Study workflow and analysis pipeline. The numbered steps correspond to those shown in the figure: (1) recruitment of postmenopausal women aged 50–70 years; (2) standardized CBCT acquisition (NewTom 3G); (3) gray value measurement in trabecular bone (OnDemand3D‐Dental); (4) fractal dimension analysis (ImageJ, box‐counting); (5) DEXA bone mineral density assessment (lumbar spine and hip); and (6) statistical analysis (SPSS) to draw conclusions.

### 2.2 CBCT Image Acquisition

All CBCT scans were obtained using a *NewTom 3G* unit (QR SRL, Verona, Italy) under standardized conditions: tube voltage of 100 kVp, tube current of 0.5 mA, exposure time of 3.6 s, field of view (FOV) of 6 inches, slice interval of 0.1 mm, and slice thickness of 0.5 mm. These parameters were kept constant across all subjects to minimize variability. Images were exported in JPEG format for subsequent analysis.

All curved and cross‐sectional reconstructions were generated using the default reconstruction tools of the NewTom software. The panoramic curve was positioned along the center of the alveolar crest using the automatic arch‐detection feature, with minor manual adjustments made by the examiner when necessary to ensure alignment with the dental arch. Cross‐sectional slices were reconstructed perpendicular to this curve. Slice thickness was fixed at 0.5 mm, and all other reconstruction parameters (kernel, window level/width, and slice interval) were kept at the device's default settings.

### 2.3 Gray Value Measurement

For gray value (GV) measurement, CBCT datasets were first converted to DICOM format and imported into *OnDemand3D‐Dental* software (CyberMed, Seoul, Korea). For each patient, six anatomical regions of interest (ROIs) within trabecular bone were identified: the anterior mandible (between the central incisors), anterior maxilla (between the central incisors), mandibular premolar region (between teeth 4 and 5), maxillary premolar region (between teeth 4 and 5), mandibular retromolar region (distal to the last molar), and maxillary tuberosity.

Within each ROI, a 25 × 25‐pixel square was selected exclusively from trabecular bone, avoiding cortical bone, tooth roots, and lamina dura wherever possible. The *Mean Gray Value* displayed by the software was recorded for each ROI. All measurements were performed by an experienced oral and maxillofacial radiologist and a trained final‐year dental student, under identical ambient lighting conditions and using the same calibrated monitor. To assess intra‐observer reliability, 20% of the images were re‐evaluated after a 2‐week interval (Figure 2).

**Figure 2:**
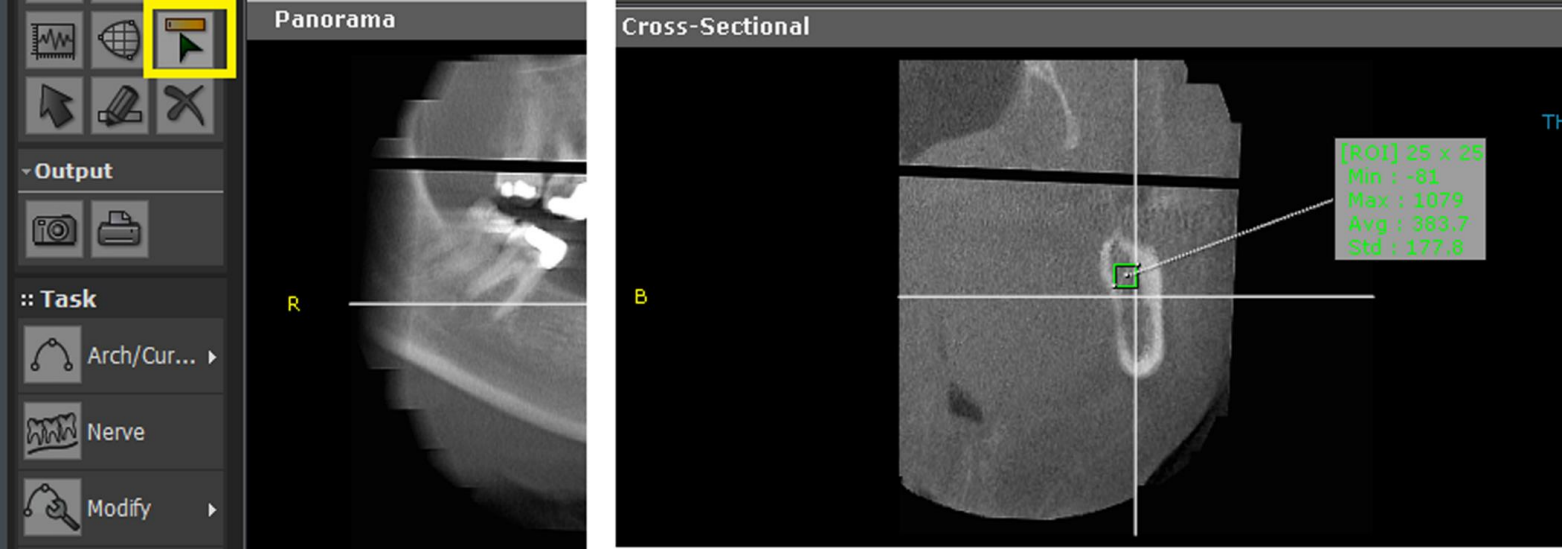
ROI selection on CBCT and extraction of Mean Gray Value (GV): the software's “Avg” gray‐level within the ROI was recorded as the GV.

### 2.4 Fractal Dimension Analysis

Fractal dimension (FD) analysis was performed using *ImageJ* software (National Institutes of Health, Bethesda, MD, USA; version 1.48 h) with the box‐counting method. The analysis process included the following steps: each ROI (25 × 25 pixels) was cropped from the CBCT slice and resized to 24 × 24 pixels to remove peripheral artifacts. A duplicate of the ROI was created, and a Gaussian blur filter with a sigma value of 35 was applied to the duplicate image to eliminate small‐ to medium‐scale brightness variations caused by differences in X‐ray attenuation and tissue thickness. The blurred image was then subtracted from the original ROI, producing a high‐pass‐filtered image in which only major density variations remained. This image was subsequently binarized, with black pixels representing trabecular bone and white pixels representing marrow spaces. The binarized image was skeletonized to extract the trabecular architecture, and FD was computed using the box‐counting function (*Analyze → Fractal Box Count*) in ImageJ. All image processing and FD measurements were performed by a single trained examiner under the supervision of a senior radiologist to ensure consistency.

### 2.5 DEXA Assessment

Bone mineral density (BMD) was measured at the lumbar spine and hip using a DEXA scanner (*OSTEOCORE*, France, 2007 model; 86 kVp, 9 mA). *T*‐scores were calculated relative to a healthy 30‐year‐old reference population of the same sex and ethnicity. Classification was performed according to WHO criteria: normal (*T*‐score ≥ −1.0), osteopenia (− 2.5 < *T*‐score < −1.0), and osteoporosis (*T*‐score ≤ −2.5) (Dimai 2017).

### 2.6 Statistical Analysis

Statistical analysis was conducted using SPSS version 21.0 (IBM Corp., Armonk, NY, USA). The normality of continuous variables was assessed using the Kolmogorov–Smirnov test. Descriptive statistics, including mean, standard deviation, and range, were calculated for all quantitative variables. Independent‐sample *t*‐tests were used to compare means between two groups, and one‐way analysis of variance (ANOVA) was applied for comparisons among more than two groups. When ANOVA indicated a significant difference, Tukey's post hoc test was applied for pairwise comparisons. Pearson's correlation coefficient was used to evaluate associations between GV, FD, and DEXA *T*‐scores. Statistical significance was set at *p* < 0.05. This study was designed as a cross‐sectional analytical investigation focused on statistical associations between CBCT‐derived parameters and DEXA *T*‐scores, rather than as a diagnostic accuracy or screening‐validation study. Accordingly, the sample size was determined for correlation analysis and is below the requirement for stable diagnostic accuracy estimation. The WHO‐based *T*‐score categories were used solely to enable comparative group analyses. Because the study was neither designed nor powered to evaluate diagnostic performance, receiver operating characteristic (ROC) analyses, sensitivity, specificity, and cut‐off determination were not performed.

## 3 Results

A total of 63 postmenopausal women met the inclusion criteria and were included in the analysis. The mean age of participants was 54.96 (8.64) years (range: 50–70 years). Based on DEXA *T*‐scores, 31 participants (49.2%) were classified as normal, 22 (34.9%) as osteopenic, and 10 (15.9%) as osteoporotic (Table 1).

**Table 1:** Age distribution according to bone density status. Values are presented as mean (SD).

Group	*n* (%)	Mean age (SD), years
Normal	31 (49.2)	54.23 (8.26)
Osteopenia	22 (34.9)	54.51 (8.70)
Osteoporosis	10 (15.9)	58.21 (9.81)
Total	63 (100)	54.96 (8.64)

Intra‐observer reliability for GV and FD measurements was excellent, with intraclass correlation coefficients (ICC) exceeding 0.70 for all ROIs.

### 3.1 *T*‐Scores by Skeletal Site

The mean lumbar spine *T*‐score was −0.91 (1.54), with subgroup means of 0.342 for normal, −1.641 for osteopenic, and −3.200 for osteoporotic individuals. The mean hip *T*‐score was 0.200 (1.048), with no participant classified as osteoporotic based on hip measurement.

### 3.2 Fractal Dimension (FD) Analysis

Mean FD values for the six evaluated ROIs are presented in Table 2. One‐way ANOVA revealed no statistically significant differences in FD values between normal and non‐normal (osteopenia + osteoporosis) groups for any ROI (all *p* > 0.05) (Figure 3).

**Table 2:** FD values by bone density status. Values are presented as mean (SD).

ROI	Normal, mean (SD)	Non‐normal, mean (SD)	*p* value
Mandibular retromolar	1.085 (0.135)	1.083 (0.152)	0.271
Mandibular premolar	1.049 (0.199)	1.048 (0.140)	0.110
Mandibular central	0.963 (0.183)	0.983 (0.167)	0.355
Maxillary premolar	1.045 (0.215)	1.075 (0.170)	0.442
Maxillary central	1.125 (0.157)	1.104 (0.133)	0.688
Maxillary tuberosity	1.081 (0.172)	1.110 (0.162)	0.353

Abbreviations: FD, fractal dimension; ROI, region of interest; SD, standard deviation.

**Figure 3:**
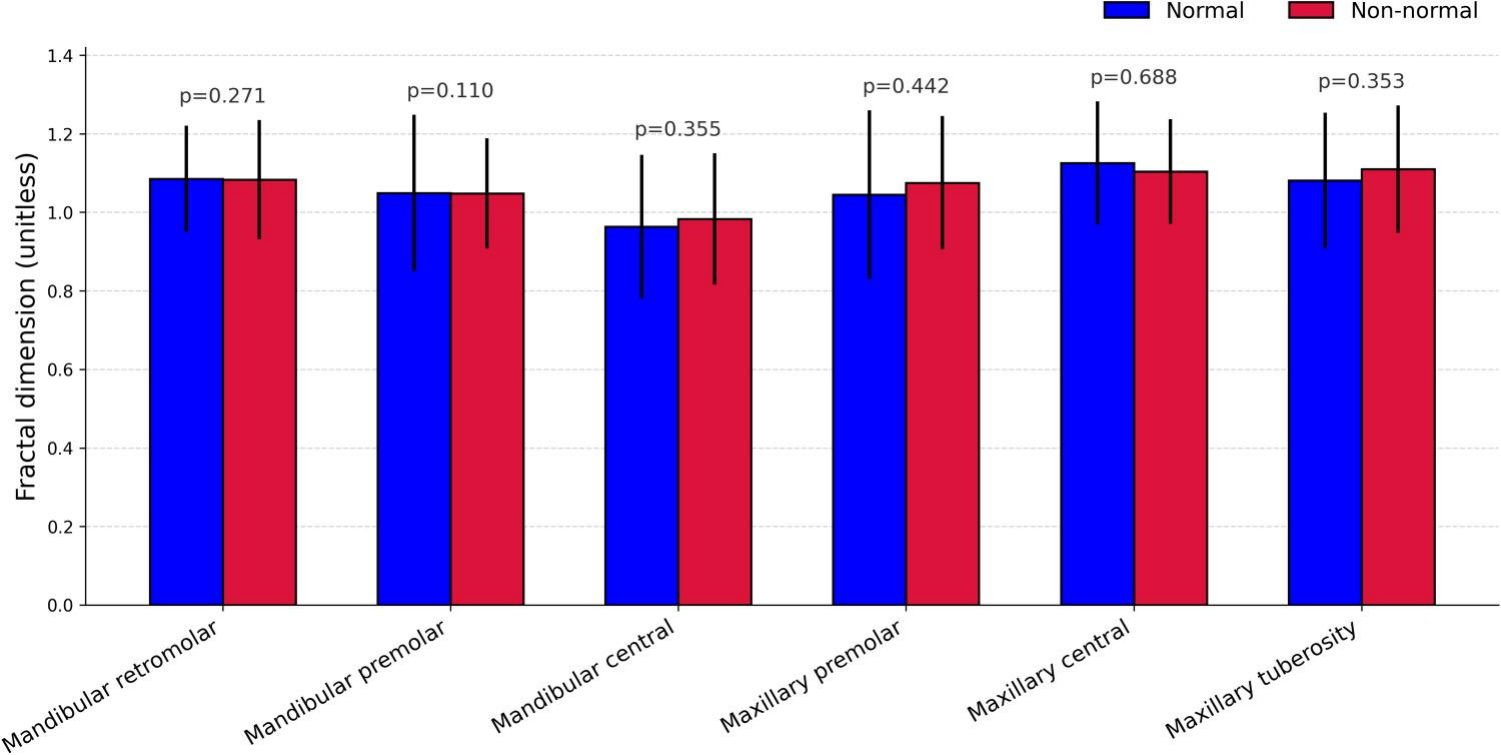
Fractal dimension by region of interest (ROI) in normal versus non‐normal BMD categories; values are presented as mean (SD). p‐values are from independent‐samples tests comparing groups within each ROI. BMD, bone mineral density; FD, fractal dimension; ROI, region of interest.

### 3.3 Gray Value (GV) Analysis

GV results are summarized in Table 3. Similar to FD, no significant differences in GV values were found between normal and non‐normal groups for any ROI (*p* > 0.05) (Figure 4).

**Table 3:** GV values by bone density status. Values are presented as mean (SD).

ROI	Normal, mean (SD)	Non‐normal, mean (SD)	*p* value
Mandibular retromolar	244.103 (102.741)	266.897 (101.326)	0.274
Mandibular premolar	346.261 (170.991)	324.553 (149.076)	0.527
Mandibular central	511.239 (215.492)	502.847 (205.160)	0.446
Maxillary premolar	304.647 (124.791)	288.275 (120.577)	0.899
Maxillary central	544.361 (144.350)	547.856 (178.828)	0.529
Maxillary tuberosity	304.674 (67.221)	288.275 (66.159)	0.516

Abbreviations: GV, gray value; ROI, region of interest; SD, standard deviation.

**Figure 4:**
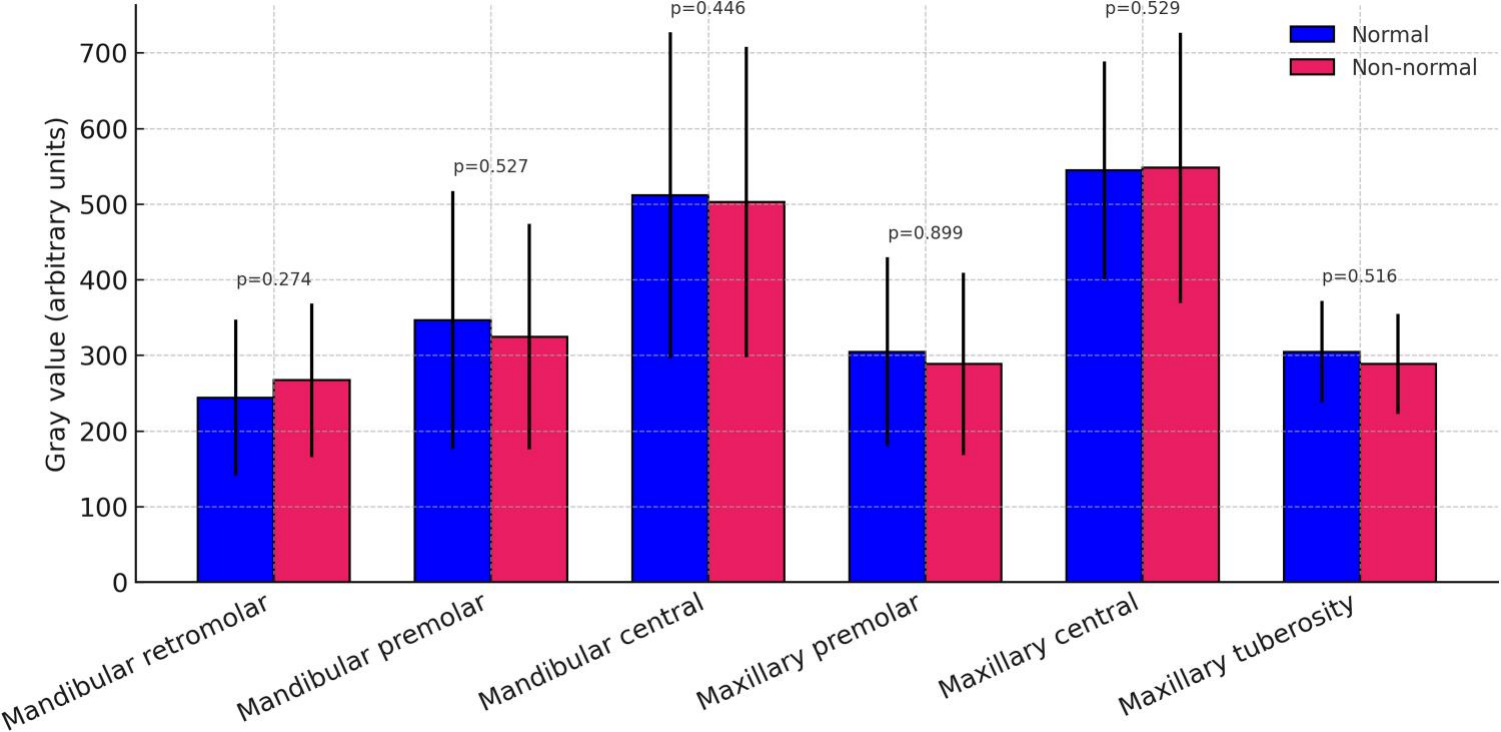
CBCT gray values by ROI in normal versus non‐normal BMD categories; values are presented as mean (SD). *p*‐values are from independent‐samples tests comparing groups within each ROI. BMD, bone mineral density; GV, gray value; ROI, region of interest.

### 3.4 Correlation Between FD and GV

Pearson's correlation analysis revealed site‐specific significant correlations.

- In the normal group, GV and FD were significantly correlated in the mandibular premolar (*p* = 0.036) and maxillary central (*p* = 0.001) regions.
- In the non‐normal group, significant correlations were found in the mandibular retromolar (*p* = 0.009) and mandibular premolar (*p* = 0.009) regions.
- In the combined dataset, significant correlations were observed in the mandibular retromolar (*p* = 0.002), mandibular premolar (*p* = 0.001), and maxillary central (*p* = 0.015) regions.

### 3.5 Correlation Between FD and *T*‐score

No significant correlations were detected between FD and lumbar spine or hip T‐scores in the normal group. However, in the non‐normal group, the FD of the maxillary premolar region was significantly correlated with lumbar spine *T*‐scores (*p* = 0.030).

## 4 Discussion

Opportunistic analysis of dental imaging obtained during routine care provides a pragmatic framework to explore associations with systemic bone mineral density (BMD) without additional exposure or cost; nevertheless, the value of image‐derived metrics remains debated (Guerra et al. 2017). In this study, we examined whether gray value (GV) and fractal dimension (FD) from cone‐beam computed tomography (CBCT) relate to dual‐energy X‐ray absorptiometry (DEXA) as a reference standard for early osteoporosis detection.

Among 63 asymptomatic postmenopausal women, 50.8% were categorized as non‐normal by lumbar spine *T*‐scores. For analysis, we aggregated osteopenic and osteoporotic cases as a single “non‐normal” category, because therapeutic decision‐making in dental practice often converges for these entities despite different T‐score bands (osteoporosis ≤ −2.5; osteopenia −2.5 to −1) (Chen, Yang, et al. 2019; Chen, Ko, et al. 2019). Our prevalence aligns with prior screenings of ostensibly healthy cohorts, in which nontrivial proportions were osteoporotic—66%, 43%, 66%, and 50% in the series of Güngör, Gulsahi, Naitoh, and Mostafa, respectively (Güngör et al. 2016; Gulsahi et al. 2010; Naitoh et al. 2007; Mostafa et al. 2016). Notably, lumbar spine BMD outperformed hip BMD for abnormality detection in our data (25.4% non‐normal by hip vs. 50.8% by lumbar), mirroring Drage (17% spine vs. 11% hip) and Barngkgei (74% by spine vs. 55% by femoral neck), suggesting earlier expression of osteoporotic change in the spine (Barngkgei et al. 2014; Drage et al. 2007). Accordingly, the lumbar T‐score was adopted as the study reference for comparison with GV and FD.

Technically, GV indexes voxel intensity on CBCT and correlates with peri‐dental bone quality; it is widely used in pre‐implant planning, with brighter voxels indicating higher GV (Pauwels et al. 2013), and its ability to proxy bone density has been increasingly substantiated (Barngkgei et al. 2014). Fractal analysis, in turn, quantifies the complexity of trabecular patterns as FD, offering a complementary descriptor of bone quality/density (Ruttimann et al. 1992; Geraets and Van Der Stelt 2000; Jolley et al. 2006; Chen et al. 2018). A key advantage is that both GV and FD can be retrospectively computed from existing CBCT datasets, facilitating retrospective association analyses without added exposure.

At the group level, no statistically significant differences were observed in mean GV or FD between the normal and non‐normal cohorts across the six jaw regions. Our principal findings were site‐specific: GV and FD correlated significantly in the retromolar and mandibular premolar regions only in the osteopenic/osteoporotic subgroup, with no such relationship in normals. These patterns are expected given the susceptibility of GV to exposure parameters, voxel size, and field of view (FOV), and the sensitivity of FD to ROI definition, FOV, and pre‐processing (Magat and Ozcan Sener 2019; Pauwels et al. 2015). The data align with Magat et al. who, across 19 ROIs, showed that although GV and FD both reflect trabecular architecture, their correlation is not uniformly significant across sites (Magat and Ozcan Sener 2019).

Regionally, only GV of the maxillary tuberosity in normals correlated with lumbar T‐scores; in non‐normals, GV–T relationships were generally absent. This is broadly consistent with Barngkgei et al. (2016), who found limited CBCT‐based differences in trabecular structure between osteoporotic and normal women, notwithstanding some local effects; note that Barngkgei used ImageJ‐based histomorphometry, whereas we analyzed site‐specific mean GV (Magat and Ozcan Sener 2019; Barngkgei et al. 2016). By contrast, Sghaireen et al. observed significant GV–density relationships in the posterior maxilla and anterior mandible, possibly reflecting their inclusion of symptomatic osteoporotic patients (Sghaireen et al. 2020).

Heterogeneity across CBCT studies is also driven by FOV: smaller FOVs increase spatial resolution and enhance visualization of the trabecular lattice, plausibly shifting measured associations with DEXA (Parsa et al. 2013; Rodrigues et al. 2015). We therefore used the smallest feasible FOV to maximize resolution in implant candidates. CBCT's vulnerability to beam‐hardening and other artifacts, and device/patient parameter variability, further complicate absolute densitometry; Campos et al. emphasized the need for parameter standardization before diagnostic application (Campos 2014).

Anatomical differences help explain why maxillary metrics often track systemic BMD more closely than mandibular metrics. Supporting prior observations, Esfahanizadeh and colleagues reported correlations of maxillary (but not mandibular) measures with femoral and spinal BMD (Esfahanizadeh et al. 2013); Southard found significant relationships between mean maxillary alveolar bone density and both lumbar and hip *T*‐scores on intraoral digital radiographs (Southard et al. 2000); and Lindh reported concordance between anterior maxillary density and lumbar BMD (Lindh et al. 2004). Physics also diverge: Lindh used CT with Hounsfield Units (HU), whereas our study used CBCT with GV (Lindh et al. 2004), and the two scales should not be treated as directly interchangeable (Kamaruddin et al. 2016). Together, these points argue for developing GV‐based thresholds/indices tailored to CBCT for jaw bone quality classification in implant planning.

Regarding FD, we observed a significant association between FD in the maxillary premolar region and lumbar T‐scores in the osteopenic/osteoporotic group, but not elsewhere. Gaalaas demonstrated site‐dependent FD values (maxilla, mandible, condyle, C2 vertebra) and supported CBCT‐based FD analysis for trabecular assessment (Gaalaas et al. 2014). Methodological choices influence outcomes: Gaalaas used coronal/axial sections and 64 × 64‐pixel ROIs, whereas, to avoid cortical plates, lamina dura, PDL space, and teeth on cross‐sectional slices, we used 25 × 25‐pixel ROIs, consistent with prior work indicating ROI size is not determinative (Gaalaas et al. 2014). Panoramic‐based studies—Yasar and Sindeaux—often found no FD differences between osteoporotic and non‐osteoporotic groups (Yaşar and Akgünlü 2006; Sindeaux et al. 2014); Koh reported FD decreases with bone loss and highlighted the mandibular premolar site on panoramics (Koh et al. 2012). In contrast, Hua found significant correlations between CBCT‐derived FD and DEXA (Hua et al. 2009). Collectively, variability in FD algorithms, imaging modality, device parameters (kVp, mA, voxel size), and ROI placement plausibly shifts results (Barngkgei et al. 2016; Hua et al. 2009; Yamamoto‐Silva et al. 2018), and Magat even noted better density prediction by panoramic‐derived FD than by CBCT‐derived FD (Magat and Ozcan Sener 2019).

A biologically plausible rationale for stronger systemic coupling in the maxilla is its greater cancellous fraction and richer vascularity (vs. thicker mandibular cortex), which may heighten sensitivity to systemic hormonal influences, whereas mandibular bone is more governed by local factors (occlusion, musculature, smoking) (Shokri et al. 2019). Thus, jaw‐based metrics are likely to remain region‐dependent, rather than uniformly indicative.

Because CBCT already underpins implant planning and other dental diagnostics, its high spatial resolution, relatively low dose, and feasibility of retrospective analysis make it a credible platform for exploring potential associations with systemic bone mineral density (Jacobs et al. 2018). Given the importance of trabecular quality for osseointegration and the frequency of edentulism in older adults, a single CBCT can serve dual purposes—procedural planning and extraction of imaging metrics for exploring associations with systemic bone mineral density—with favorable cost–utility (Norman et al. 2008; Ibrahim et al. 2013). A systematic review by Guerra et al. signals diagnostic value of CBCT for identifying low‐BMD patients, while underscoring the need for optimized device settings and measurement protocols (Guerra et al. 2017). Among FD methods in ImageJ, box (tile) counting remains prevalent for its simplicity and was used here (Ilhan et al. 2015). Complementing our trabecular‐focused metrics (GV and FD), a contemporary BMC Medical Imaging study reported that CBCT‐derived cortical/radiomorphometric indices (CTMI, CTI(S), CTI(I), CTCI) correlate with DEXA T‐scores and discriminate groups; notably, CTMI showed the strongest separation, and a simple MLP reached ≈75% diagnostic accuracy—supporting the idea that combining cortical indices with GV/FD may further enhance performance (Rafieizadeh et al. 2025).

Strengths include a standardized imaging protocol, small FOV, carefully defined ROIs on cross‐sectional slices, and a consistent reference (lumbar *T*‐score). Limitations are CBCT's inter‐device GV variability and artifact susceptibility, and FD's sensitivity to ROI and preprocessing. Importantly, CBCT gray values are not standardized and, unlike Hounsfield units in medical CT, are influenced by scatter radiation, field of view, exposure parameters, and device‐specific reconstruction algorithms; they should therefore be interpreted as relative rather than absolute indicators of bone density. The relatively small sample, and in particular the limited number of osteoporotic participants (*n* = 10), reduced statistical power for subgroup comparisons. In addition, multiple site‐ and group‐specific correlations were examined without formal correction for multiple comparisons; the observed site‐specific associations should therefore be regarded as exploratory and hypothesis‐generating rather than confirmatory. The cross‐sectional design precludes inference on temporal change; larger prospective, multicenter studies are warranted to confirm these associations and to clarify the potential value of CBCT‐derived metrics in relation to systemic bone status.

Given the observed correlations between CBCT‐derived parameters and DEXA T‐scores in the present study, these findings should be interpreted as preliminary and exploratory evidence that warrants further investigation in future research with more robust study designs. In this context, future studies are encouraged to evaluate the potential diagnostic performance of these imaging biomarkers using larger, adequately powered samples and validated methodological frameworks. In particular, the application of advanced analytical approaches, including machine‐learning models, receiver operating characteristic (ROC) curve analysis, and optimal threshold determination, may provide more definitive evidence regarding their diagnostic utility; for example, deep‐learning models have already been applied to estimate bone mineral density from quantitative CBCT images (Park et al. 2023). Furthermore, both internal validation strategies (e.g., cross‐validation or bootstrapping) and external validation in independent populations are essential to assess the stability, reproducibility, and generalizability of any proposed models. Such comprehensive validation frameworks are necessary before any clinical translation or application in opportunistic screening of systemic bone mineral status can be considered.

## 5 Conclusion

In this cohort of 63 postmenopausal women, there were no group‐level differences in mean GV or FD between normal and non‐normal BMD categories across six jaw regions; however, site‐specific patterns emerged. Significant GV–FD coupling was confined to the mandibular retromolar and premolar sites among osteopenic/osteoporotic participants, maxillary tuberosity GV correlated with lumbar T‐scores in normals, and maxillary premolar FD correlated with lumbar T‐scores in the non‐normal group. Lumbar spine BMD also proved more sensitive than hip BMD for detecting abnormality. Collectively, these findings suggest that CBCT‐derived metrics may reflect localized trabecular bone characteristics and show limited, region‐dependent associations with systemic bone mineral density. However, given the cross‐sectional design and limited sample size, these parameters cannot be considered diagnostic or screening tools, and further large‐scale studies are required to determine their clinical utility in osteoporosis assessment.

Clinically, we recommend incorporating standardized GV and FD extraction from prespecified ROIs (mandibular premolar/retromolar and maxillary premolar/tuberosity) into CBCT workflows used for implant planning. To strengthen reproducibility and generalizability, harmonized acquisition parameters (small FOV, fixed voxel size, and kVp/mA), reproducible ROI templates, and development of CBCT‐specific GV thresholds are needed. Emerging evidence further suggests that combining cortical/radiomorphometric indices with GV/FD may enhance diagnostic performance; this should be evaluated in prospective, multicenter studies with inter‐device calibration and sensitivity analyses for artifacts. In sum, a single CBCT may serve a dual purpose—procedural planning and, as a source of imaging metrics, the exploration of potential associations with systemic bone mineral density—and may, in future studies, help identify patients who could benefit from further evaluation with DEXA, although its diagnostic validity for this purpose has not yet been established.

## Author Contributions

Golnoush Saeidi and Mohammad Mahdi Maleki contributed to data collection, literature review, manuscript writing, and preparation of the final draft. Abbas Shokri conceptualized and designed the study, developed the methodology, supervised all stages of the research, and provided critical revision of the manuscript as the principal supervisor. Leili Tapak performed the statistical analysis, interpreted the quantitative data, and contributed to the methodology of data analysis. All authors have read and approved the final version of the manuscript.

## Funding

The authors have nothing to report.

## Ethics Statement

This study adheres to the ethical and legal considerations of research, follows the principles and guidelines of the International Committee on Publication Ethics (COPE), and is approved by the Research Ethics Committees of Hamadan University of Medical Sciences with ethics code IR.UMSHA.REC.1398.711, and informed consent was obtained from all participants prior to participation in the study.

## Consent

All of the authors of this paper affirm that it has been submitted solely to this journal and that it is not concurrently under consideration for publication in another journal. All of the named authors have been involved in the work leading to the publication of the paper and have read the paper before it was submitted for publication.

## Conflicts of Interest

The authors declare no conflicts of interest.

## Data Availability

The data that support the findings of this study are available from the corresponding author upon reasonable request.

## References

[cre270474-bib-0001] Adler, R. A. 2020. Osteoporosis in Men. Osteoporosis, 391–406. Springer.

[cre270474-bib-0002] Barngkgei, I. , I. Al Haffar , and R. Khattab . 2014. “Osteoporosis Prediction From the Mandible Using Cone‐Beam Computed Tomography.” Imaging Science in Dentistry 44, no. 4: 263–271. 10.5624/isd.2014.44.4.263 PMC4245467 25473633

[cre270474-bib-0003] Barngkgei, I. , I. Al Haffar , E. Shaarani , R. Khattab , and A. Mashlah . 2016. “Assessment of Jawbone Trabecular Bone Structure Amongst Osteoporotic Women by Cone‐Beam Computed Tomography: The Osteosyr Project.” Journal of Investigative and Clinical Dentistry 7, no. 4: 332–340. 10.1111/jicd.12170 26097193

[cre270474-bib-0004] Black, D. M. , and C. J. Rosen . 2016. “Postmenopausal Osteoporosis.” New England Journal of Medicine 374, no. 3: 254–262. 10.1056/NEJMcp1513724 26789873

[cre270474-bib-0005] Bushong, S. C. 2013. Radiologic Science for Technologists‐E‐Book: Physics, Biology, and Protection. Elsevier Health Sciences.

[cre270474-bib-0006] Campos, M. J. S. 2014. “Bone Mineral Density in Cone Beam Computed Tomography: Only a Few Shades of Gray.” World Journal of Radiology 6, no. 8: 607–612. 10.4329/wjr.v6.i8.607 PMC4147441 25170398

[cre270474-bib-0007] Carvalho, B. F. , J. G. K. de Castro , N. S. de Melo , et al. 2022. “Fractal Dimension Analysis on CBCT Scans for Detecting Low Bone Mineral Density in Postmenopausal Women.” Imaging Science in Dentistry 52, no. 1: 53–60. 10.5624/isd.20210172 PMC8967487 35387102

[cre270474-bib-0008] Cassetta, M. , L. V. Stefanelli , A. Pacifici , L. Pacifici , and E. Barbato . 2014. “How Accurate Is CBCT in Measuring Bone Density? A Comparative CBCT‐CT In Vitro Study.” Clinical Implant Dentistry and Related Research 16, no. 4: 471–478. 10.1111/cid.12027 23294461

[cre270474-bib-0009] de Castro, J. G. K. , B. F. Carvalho , N. S. de Melo , et al. 2020. “A New Cone‐Beam Computed Tomography–Driven Index for Osteoporosis Prediction.” Clinical Oral Investigations 24: 3193–3202. 10.1007/s00784-019-03193-4 31912243

[cre270474-bib-0010] Chen, J. S. C. , A. Yang , and D. F. Murrell . 2019. “Prevalence and Pathogenesis of Osteopenia and Osteoporosis in Epidermolysis Bullosa: An Evidence‐Based Review.” Experimental Dermatology 28, no. 10: 1122–1130. 10.1111/exd.13771 30118182

[cre270474-bib-0011] Chen, L.‐R. , N.‐Y. Ko , and K.‐H. Chen . 2019. “Medical Treatment for Osteoporosis: From Molecular to Clinical Opinions.” International Journal of Molecular Sciences 20, no. 9: 2213. 10.3390/ijms20092213 PMC6540613 31064048

[cre270474-bib-0012] Chen, Q. , N. Bao , Q. Yao , and Z.‐Y. Li . 2018. “Fractal Dimension: A Complementary Diagnostic Indicator of Osteoporosis to Bone Mineral Density.” Medical Hypotheses 116: 136–138. 10.1016/j.mehy.2018.05.006 29857898

[cre270474-bib-0013] Cummings, S. R. , J. L. Kelsey , M. C. Nevitt , and K. J. O'dowd . 1985. “Epidemiology of Osteoporosis and Osteoporotic Fractures.” Epidemiologic Reviews 7, no. 1: 178–208. 10.1093/oxfordjournals.epirev.a036281 3902494

[cre270474-bib-0014] Dimai, H. P. 2017. “Use of Dual‐Energy X‐Ray Absorptiometry (DXA) for Diagnosis and Fracture Risk Assessment; WHO‐Criteria, T‐and Z‐Score, and Reference Databases.” Bone 104: 39–43. 10.1016/j.bone.2016.12.016 28041872

[cre270474-bib-0015] Drage, N. A. , R. M. Palmer , G. Blake , R. Wilson , F. Crane , and I. Fogelman . 2007. “A Comparison of Bone Mineral Density in the Spine, Hip and Jaws of Edentulous Subjects.” Clinical Oral Implants Research 18, no. 4: 496–500. 10.1111/j.1600-0501.2007.01379.x 17517057

[cre270474-bib-0016] Esfahanizadeh, N. , S. Davaie , A. R. Rokn , et al. 2013. “Correlation Between Bone Mineral Density of Jaws and Skeletal Sites in an Iranian Population Using Dual X‐Ray Energy Absorptiometry.” Dental Research Journal 10, no. 4: 460–466. PMC3793408 24130580

[cre270474-bib-0017] Gaalaas, L. , L. Henn , P. R. Gaillard , M. Ahmad , and M. S. Islam . 2014. “Analysis of Trabecular Bone Using Site‐Specific Fractal Values Calculated From Cone Beam CT Images.” Oral Radiology 30, no. 2: 179–185.

[cre270474-bib-0018] Gambacciani, M. 2020. “The Relative Contributions of Menopause and Aging to Postmenopausal Osteoporosis.” Climacteric 23, no. 1: 105. 10.1080/13697137.2019.1679114 31709834

[cre270474-bib-0019] Geraets, W. G. , and P. F. Van Der Stelt . 2000. “Fractal Properties of Bone.” Dentomaxillofacial Radiology 29, no. 3: 144–153. 10.1038/sj/dmfr/4600524 10849540

[cre270474-bib-0020] Guerra, E. N. , F. T. Almeida , F. V. Bezerra , et al. 2017. “Capability of Cbct to Identify Patients With Low Bone Mineral Density: A Systematic Review.” Dentomaxillofacial Radiology 46, no. 8: 20160475. 10.1259/dmfr.20160475 PMC5965944 28555506

[cre270474-bib-0021] Gulsahi, A. , C. Paksoy , S. Ozden , N. Kucuk , A. Cebeci , and Y. Genc . 2010. “Assessment of Bone Mineral Density in the Jaws and Its Relationship to Radiomorphometric Indices.” Dentomaxillofacial Radiology 39, no. 5: 284–289. 10.1259/dmfr/20522657 PMC3520251 20587652

[cre270474-bib-0022] Güngör, E. , D. Yildirim , and R. Çevik . 2016. “Evaluation of Osteoporosis in Jaw Bones Using Cone Beam CT and Dual‐Energy X‐Ray Absorptiometry.” Journal of Oral Science 58, no. 2: 185–194. 10.2334/josnusd.15-0609 27349539

[cre270474-bib-0023] Horner, K. , K. Karayianni , A. Mitsea , et al. 2007. “The Mandibular Cortex on Radiographs as a Tool for Osteoporosis Risk Assessment: The Osteodent Project.” Journal of Clinical Densitometry 10, no. 2: 138–146. 10.1016/j.jocd.2007.02.004 17449308

[cre270474-bib-0024] Hua, Y. , O. Nackaerts , J. Duyck , F. Maes , and R. Jacobs . 2009. “Bone Quality Assessment Based on Cone Beam Computed Tomography Imaging.” Clinical Oral Implants Research 20, no. 8: 767–771. 10.1111/j.1600-0501.2008.01677.x 19489931

[cre270474-bib-0025] Ibrahim, N. , A. Parsa , B. Hassan , P. Van der Stelt , and D. Wismeijer . 2013. “Diagnostic Imaging of Trabecular Bone Microstructure for Oral Implants: A Literature Review.” Dentomaxillofacial Radiology 42, no. 3: 20120075. 10.1259/dmfr.20120075 PMC3667540 23420864

[cre270474-bib-0026] Ilhan, B. , A. Saraçoglu , H. Koca , H. Boyacioglu , and P. Güneri . 2015. “A Comparison of Fractal Dimension Values of Peri‐Implant Bone and Healthy Contralateral Side Using Panoramic Radiographs.” Journal of Oral and Maxillofacial Radiology 3, no. 1: 1.

[cre270474-bib-0027] Isayev, A. , N. Velieva , L. Isedisha , Z. Isayeva , K. Kamburoğlu , and F. Kuyumcu . 2023. “Cone‐Beam Computed Tomography as a Prediction Tool for Osteoporosis in Postmenopausal Women: A Systematic Literature Review.” Diagnostics 13, no. 6: 1027. 10.3390/diagnostics13061027 PMC10047831 36980335

[cre270474-bib-0028] Jacobs, R. , B. Salmon , M. Codari , B. Hassan , and M. M. Bornstein . 2018. “Cone Beam Computed Tomography in Implant Dentistry: Recommendations for Clinical Use.” BMC Oral health 18, no. 1: 88. 10.1186/s12903-018-0523-5 PMC5952365 29764458

[cre270474-bib-0029] Jolley, L. , S. Majumdar , and S. Kapila . 2006. “Technical Factors in Fractal Analysis of Periapical Radiographs.” Dentomaxillofacial Radiology 35, no. 6: 393–397. 10.1259/dmfr/30969642 17082328

[cre270474-bib-0030] Kamaruddin, N. , Rajion, Z. A. , Yusof, A. , and Aziz, M. E. , ed. 2016. Relationship Between Hounsfield Unit in CT Scan and Gray Scale in CBCT. AIP Conference Proceedings. AIP Publishing LLC.

[cre270474-bib-0031] Koh, K.‐J. , H.‐N. Park , and K.‐A. Kim . 2012. “Prediction of Age‐Related Osteoporosis Using Fractal Analysis on Panoramic Radiographs.” Imaging Science in Dentistry 42, no. 4: 231–235. 10.5624/isd.2012.42.4.231 PMC3534177 23301209

[cre270474-bib-0032] Lindh, C. , K. Obrant , and A. Petersson . 2004. “Maxillary Bone Mineral Density and Its Relationship to the Bone Mineral Density of the Lumbar Spine and Hip.” Oral Surgery, Oral Medicine, Oral Pathology, Oral Radiology, and Endodontology 98, no. 1: 102–109. 10.1016/s1079-2104(03)00460-8 15243479

[cre270474-bib-0033] Lindsay, R. , F. Cosman , E. B. Osteoporosis , A. Fauci , and K. Isselbacher . 2001. Harrison's Principles of Internal Medicine. McGraw‐Hill.

[cre270474-bib-0034] Lopes, R. , and N. Betrouni . 2009. “Fractal and Multifractal Analysis: A Review.” Medical Image Analysis 13, no. 4: 634–649. 10.1016/j.media.2009.05.003 19535282

[cre270474-bib-0035] Lucas, K. , I. Nolte , V. Galindo‐Zamora , et al. 2017. “Comparative Measurements of Bone Mineral Density and Bone Contrast Values in Canine Femora Using Dual‐Energy X‐Ray Absorptiometry and Conventional Digital Radiography.” BMC Veterinary Research 13, no. 1: 130. 10.1186/s12917-017-1047-y PMC5426025 28490330

[cre270474-bib-0036] Magat, G. , and S. Ozcan Sener . 2019. “Evaluation of Trabecular Pattern of Mandible Using Fractal Dimension, Bone Area Fraction, and Gray Scale Value: Comparison of Cone‐Beam Computed Tomography and Panoramic Radiography.” Oral Radiology 35, no. 1: 35–42. 10.1007/s11282-018-0316-1 30484179

[cre270474-bib-0037] Marcus, R. , and S. Majumder . 1996. “The Nature of Osteoporosis.” Osteoporosis 1: 3–17.

[cre270474-bib-0038] Mostafa, R. A. , E. A. Arnout , and M. M. Abo el‐Fotouh . 2016. “Feasibility of Cone Beam Computed Tomography Radiomorphometric Analysis and Fractal Dimension in Assessment of Postmenopausal Osteoporosis in Correlation With Dual X‐Ray Absorptiometry.” Dentomaxillofacial Radiology 45, no. 7: 20160212. 10.1259/dmfr.20160212 PMC5606262 27418348

[cre270474-bib-0039] Nackaerts, O. , R. Jacobs , H. Devlin , et al. 2008. “Osteoporosis Detection Using Intraoral Densitometry.” Dentomaxillofacial Radiology 37, no. 5: 282–287. 10.1259/dmfr/30424604 18606750

[cre270474-bib-0040] Naitoh, M. , Y. Kurosu , K. Inagaki , A. Katsumata , T. Noguchi , and E. Ariji . 2007. “Assessment of Mandibular Buccal and Lingual Cortical Bones in Postmenopausal Women.” Oral Surgery, Oral Medicine, Oral Pathology, Oral Radiology, and Endodontology 104, no. 4: 545–550. 10.1016/j.tripleo.2007.04.034 17689117

[cre270474-bib-0041] Norman, J. , J. G. Shapter , K. Short , L. J. Smith , and N. L. Fazzalari . 2008. “Micromechanical Properties of Human Trabecular Bone: A Hierarchical Investigation Using Nanoindentation.” Journal of Biomedical Materials Research. Part A 87, no. 1: 196–202. 10.1002/jbm.a.31766 18085652

[cre270474-bib-0042] Park, C.‐S. , S.‐R. Kang , J.‐E. Kim , et al. 2023. “Validation of Bone Mineral Density Measurement Using Quantitative CBCT Image Based on Deep Learning.” Scientific Reports 13, no. 1: 11921. 10.1038/s41598-023-38943-8 PMC10366160 37488135

[cre270474-bib-0043] Parsa, A. , N. Ibrahim , B. Hassan , A. Motroni , P. Van der Stelt , and D. Wismeijer . 2013. “Influence of Cone Beam CT Scanning Parameters on Grey Value Measurements at an Implant Site.” Dentomaxillofacial Radiology 42, no. 3: 79884780. 10.1259/dmfr/79884780 PMC3667541 22933535

[cre270474-bib-0044] Paschalis, E. P. , P. Fratzl , S. Gamsjaeger , et al. 2016. “Aging Versus Postmenopausal Osteoporosis: Bone Composition and Maturation Kinetics at Actively‐Forming Trabecular Surfaces of Female Subjects Aged 1 to 84 Years.” Journal of Bone and Mineral Research 31, no. 2: 347–357. 10.1002/jbmr.2696 26308158

[cre270474-bib-0045] Pauwels, R. , T. Faruangsaeng , T. Charoenkarn , N. Ngonphloy , and S. Panmekiate . 2015. “Effect of Exposure Parameters and Voxel Size on Bone Structure Analysis in CBCT.” Dentomaxillofacial Radiology 44, no. 8: 20150078. 10.1259/dmfr.20150078 PMC4628422 26054572

[cre270474-bib-0046] Pauwels, R. , O. Nackaerts , N. Bellaiche , et al. 2013. “Variability of Dental Cone Beam CT Grey Values for Density Estimations.” British Journal of Radiology 86, no. 1021: 20120135. 10.1259/bjr.20120135 PMC4651064 23255537

[cre270474-bib-0047] Pickhardt, P. J. , L. J. Lee , A. Muñoz del Rio , et al. 2011. “Simultaneous Screening for Osteoporosis at CT Colonography: Bone Mineral Density Assessment Using MDCT Attenuation Techniques Compared With the DXA Reference Standard.” Journal of Bone and Mineral Research 26, no. 9: 2194–2203. 10.1002/jbmr.428 PMC3304444 21590738

[cre270474-bib-0048] Rachner, T. D. , S. Khosla , and L. C. Hofbauer . 2011. “Osteoporosis: Now and the Future.” Lancet 377, no. 9773: 1276–1287. 10.1016/S0140-6736(10)62349-5 PMC3555696 21450337

[cre270474-bib-0049] Rafieizadeh, S. , S. Lari , M. M. Maleki , A. Shokri , and L. Tapak . 2025. “Investigation of the Correlation Between Radiomorphometric Indices in Cone‐Beam Computed Tomography Images and Dual X‐Ray Absorptiometry Bone Density Test Results in Postmenopausal Women.” BMC Medical Imaging 25, no. 1: 203. 10.1186/s12880-025-01739-5 PMC12139361 40474111

[cre270474-bib-0050] Rodrigues, A. F. , M. J. da Silva Campos , A. Chaoubah , M. R. Fraga , and R. W. F. Vitral . 2015. “Use of Gray Values in CBCT and MSCT Images for Determination of Density: Influence of Variation of FOV Size.” Implant Dentistry 24, no. 2: 155–159. 10.1097/ID.0000000000000179 25706259

[cre270474-bib-0051] Ruttimann, U. E. , R. L. Webber , and J. B. Hazelrig . 1992. “Fractal Dimension From Radiographs of Peridental Alveolar Bone.” Oral Surgery, Oral Medicine, Oral Pathology 74, no. 1: 98–110. 10.1016/0030-4220(92)90222-c 1508517

[cre270474-bib-0052] Sghaireen, M. G. , K. K. Ganji , M. K. Alam , et al. 2020. “Comparing the Diagnostic Accuracy of CBCT Grayscale Values With DXA Values for the Detection of Osteoporosis.” Applied Sciences 10, no. 13: 4584.

[cre270474-bib-0053] Shokri, A. , M. Ghanbari , F. H. Maleki , L. Ramezani , P. Amini , and L. Tapak . 2019. “Relationship of Gray Values in Cone Beam Computed Tomography and Bone Mineral Density Obtained by Dual Energy X‐Ray Absorptiometry.” Oral Surgery, Oral Medicine, Oral Pathology and Oral Radiology 128, no. 3: 319–331. 10.1016/j.oooo.2019.04.017 31171482

[cre270474-bib-0054] Shrout, M. K. , C. F. Hildebolt , and B. J. Potter . 1997. “The Effect of Varying the Region of Interest on Calculations of Fractal Index.” Dentomaxillofacial Radiology 26, no. 5: 295–298. 10.1038/sj.dmfr.4600260 9482002

[cre270474-bib-0055] Sindeaux, R. , P. T. S. Figueiredo , N. S. de Melo , et al. 2014. “Fractal Dimension and Mandibular Cortical Width in Normal and Osteoporotic Men and Women.” Maturitas 77, no. 2: 142–148. 10.1016/j.maturitas.2013.10.011 24289895

[cre270474-bib-0056] Slaidina, A. , E. Nikitina , A. Abeltins , U. Soboleva , and A. Lejnieks . 2022. “Gray Values of the Cervical Vertebrae Detected by Cone Beam Computed Tomography for the Identification of Osteoporosis and Osteopenia in Postmenopausal Women.” Oral Surgery, Oral Medicine, Oral Pathology and Oral Radiology 133, no. 1: 100–109. 10.1016/j.oooo.2021.06.014 34535433

[cre270474-bib-0057] Southard, K. A. , T. E. Southard , J. A. Schlechte , and P. A. Meis . 2000. “The Relationship Between the Density of the Alveolar Processes and That of Post‐Cranial Bone.” Journal of Dental Research 79, no. 4: 964–969. 10.1177/00220345000790041201 10831099

[cre270474-bib-0058] Sridevi, A. , and V. Ragi . 2016. “Management of Osteoporosis in Women—A Prevalence and Interventional Study.” International Archives of Integrated Medicine 3: 140–145.

[cre270474-bib-0059] Tafraouti, A. , Hassouni, M. E. , Toumi, H. , Lespessailles, E. , and Jennane, R. 2014. “Osteoporosis Diagnosis Using Fractal Analysis and Support Vector Machine.” 2014 Tenth International Conference on Signal‐Image Technology and Internet‐Based Systems; November 23–27.

[cre270474-bib-0060] Tsiklakis, K. , K. Syriopoulos , and H. Stamatakis . 2004. “Radiographic Examination of the Temporomandibular Joint Using Cone Beam Computed Tomography.” Dentomaxillofacial Radiology 33, no. 3: 196–201. 10.1259/dmfr/27403192 15371321

[cre270474-bib-0061] De Vos, W. , J. Casselman , and G. R. J. Swennen . 2009. “Cone‐Beam Computerized Tomography (CBCT) Imaging of the Oral and Maxillofacial Region: A Systematic Review of the Literature.” International Journal of Oral and Maxillofacial Surgery 38, no. 6: 609–625. 10.1016/j.ijom.2009.02.028 19464146

[cre270474-bib-0062] Walker, M. D. , J. Williams , S. K. Lewis , J. C. Bai , B. Lebwohl , and P. H. R. Green . 2020. “Measurement of Forearm Bone Density by Dual Energy X‐Ray Absorptiometry Increases the Prevalence of Osteoporosis in Men With Celiac Disease.” Clinical Gastroenterology and Hepatology 18, no. 1: 99–106. 10.1016/j.cgh.2019.03.049 30981003

[cre270474-bib-0063] Winzenberg, T. , and G. Jones . 2011. “Dual Energy X‐Ray Absorptiometry.” Australian Family Physician 40, no. 1/2: 43–44. 21301693

[cre270474-bib-0064] Yamamoto‐Silva, F. P. , C. F. de Oliveira Siqueira , M. A. G. S. Silva , et al. 2018. “Influence of Voxel Size on Cone‐Beam Computed Tomography‐Based Detection of Vertical Root Fractures in the Presence of Intracanal Metallic Posts.” Imaging Science in Dentistry 48, no. 3: 177–184. 10.5624/isd.2018.48.3.177 PMC6148034 30276154

[cre270474-bib-0065] Yaşar, F. , and F. Akgünlü . 2006. “The Differences in Panoramic Mandibular Indices and Fractal Dimension Between Patients With and Without Spinal Osteoporosis.” Dentomaxillofacial Radiology 35, no. 1: 1–9. 10.1259/dmfr/97652136 16421256

